# *Milnesium berladnicorum* sp. n. (Eutardigrada, Apochela, Milnesiidae), a new species of water bear from Romania

**DOI:** 10.3897/zookeys.429.7755

**Published:** 2014-07-29

**Authors:** Daniel Adrian Ciobanu, Krzysztof Zawierucha, Ioan Moglan, Łukasz Kaczmarek

**Affiliations:** 1 Faculty of Biology, Alexandru Ioan Cuza University of Iași, B-dul Carol I, no. 20A, 700505 Iași, Romania; 2 Department of Animal Taxonomy and Ecology, Faculty of Biology, A. Mickiewicz University in Poznań, Umultowska 89, 61-614 Poznań, Poland; 3 Prometeo researcher, Laboratorio de Ecología Natural y Aplicada de Invertebrados,Universidad Estatal Amazónica, Puyo, Ecuador

**Keywords:** Europe, new species, Palearctic, Tardigrada, taxonomy

## Abstract

In a lichen sample collected from a tree in Bârlad town (Vaslui County, Romania), a new tardigrade species belonging to the genus *Milnesium* (*granulatum* group) was found. *Milnesium berladnicorum* **sp. n.** is most similar (in the type of dorsal sculpture) to *Milnesium beasleyi* Kaczmarek et al., 2012 but differs from it mainly by having a different claw configuration and some morphometric characters. Additionally, the new species differs from other congeners of the *granulatum* group by the different type of dorsal sculpture, claw configuration and some morphometric characters.

## Introduction

In Romania, studies on tardigrades were rather fragmentary and with a significant discontinuity in time (last 40 years). Even though during this period more than 150 taxa (species and subspecies) were reported for this region (Rudescu 1964), many of them are now considered as non-valid, have been synonymized, or require confirmation. In fact, in the light of modern taxonomy, only 127 tardigrades species are consider to be present in Romania (in 26 of 41 Romanian counties) (Ciobanu et al. 2014). Species from the genus *Milnesium* Doyère, 1840 are known from many localities throughout the world, from the Antarctic through tropical and temperate zones to the Arctic regions (e.g. Tumanov 2006; Kaczmarek et al. 2012a, 2012b; Michalczyk et al. 2012a). Since the genus was recently re-described (Michalczyk et al. 2012a, 2012b) new records and species have been reported from various localities (e.g. Kaczmarek et al. 2012a; Meyer et al. 2013; Zawierucha et al. 2014; Ciobanu et al. 2014). Taking into consideration that some morphological characters were omitted in older records of *Milnesium* specimens, all such records should be verified (Michalczyk et al. 2012a, 2012b). Until now in Romania only three *Milnesium* species have been reported: *Milnesium tardigradum* sensu lato Doyère, 1840, *Milnesium granulatum* (Ramazzotti, 1962) and *Milnesium asiaticum* Tumanov, 2006, but all early records of the first species should be verified (Michalczyk et al. 2012a, 2012b; Ciobanu et al. 2014). In this paper a new species of the genus *Milnesium* is described and illustrated.

## Materials and methods

In a lichen sample collected by the first author in Bârlad town in July, 2013, 53 individuals and two exuvia (with 16 eggs) of the new species were found. Additionally, 55 specimens of *Ramazzottius oberhaeuseri* (Doyère, 1840) were found in the same sample, including 9 specimens in simplex stage and 9 eggs.

All specimens were extracted according to Dastych (1980, 1985) and mounted on microscope slides in Hoyer’s medium. Observations, measurements and photomicrographs were taken using Phase Contrast Microscopy (PCM) (Olympus BX41 with digital camera ARTCAM-300Mi). All measurements (determined with QuickPhoto Camera 2.3) are given in micrometers [μm].

Body length was measured from the mouth to the end of the body excluding the hind legs. The buccal tube and claws characteristics were measured according to Tumanov (2006) and Michalczyk et al. (2012a). Subsequently, claw configuration is described according to Michalczyk et al. (2012a, 2012b). Other morphometric data were calculated using the *pt* ratio: the ratio of the length of a given structure to the length of the buccal tube, expressed as a percentage (Pilato 1981). The *pt* values are always provided in *italics* in order to differentiate them from length values.

Characteristics and measurements of the species used in the differential diagnosis are given according to the original descriptions (Ramazzotti 1962; Pilato et al. 2002; Kaczmarek et al. 2004; Tumanov 2006; Kaczmarek and Michalczyk 2007; Wallendorf and Miller 2009; Kaczmarek et al. 2012a; Meyer et al. 2013) or are based on direct examination of type material (holotype and paratypes of *Milnesium beasleyi* Kaczmarek et al. 2012a). *Ramazzottius* specimens were verified and identified using the key to the World Tardigrada (Ramazzotti and Maucci 1983), a more modern key to the genus *Ramazzottius* (Biserov 1998), and remarks discussed by Pilato et al. (2013).

Morphometric data were handled using the ''Apochela'' ver. 1.1 template available from the Tardigrada Register (Michalczyk and Kaczmarek 2013). Raw data underlying the description of *Milnesium berladnicorum* sp. n. are deposited in the Tardigrada Register under http://www.tardigrada.net/register/0014.htm

## Results

### Taxonomic Account
Phylum: Tardigrada Doyère, 1840
Class: Eutardigrada Richters, 1926
Order: Apochela Schuster, Nelson, Grigarick and Christenberry, 1980
Family: Milnesiidae Ramazzotti, 1962
Genus: *Milnesium* Doyère, 1840

#### Milnesium berladnicorum sp. n.

Taxon classificationAnimaliaApochelaMilnesiidae

http://zoobank.org/FBF8C785-2B53-48B2-B696-D442BAD89A0F

http://www.tardigrada.net/register/0014.htm

Figs 1 –6 , Table 1

##### Material examined.

Holotype (female), 52 paratypes and 2 exuvia with 7 and 9 smooth eggs.

##### Description

**(measurements and statistics in Table 1).** Body brownish (in live specimens) or transparent (in fixed specimens) with eyes (visible before and after mounting in Hoyer’s medium - 90% of fixed specimens had eyes). Six peribuccal papillae (ventral papilla smallest) and six peribuccal lamellae (of equal size) around the mouth opening present. Two cephalic papillae positioned laterally. The cuticle is covered with numerous tiny, shallow and rounded depressions (pseudopores) (Figs 4–5). Under PCM these pseudopores are visible as light spots, placing the species within the *granulatum* group. Bucco-pharyngeal apparatus of the *Milnesium* type (Fig. 6). Buccal tube funnel-shaped, wider anteriorly (on average the posterior diameter is 73% of the anterior diameter). Pharyngeal bulb elongated, pear-shaped and without placoids or septulum. Claws of the *Milnesium* type, slender (Figs 2–3). Primary branches on all legs with small accessory points on the top of the branch. Secondary claws of all legs with rounded basal thickenings (lunules) (sometimes barely visible) (Fig. 3). Secondary branches of external claws I–III and posterior and anterior claws IV with two points. Secondary branches of internal claws I–III with three points (i.e. claw configuration: [2-3]-[2-2]) (Figs 2–3). Single, long transverse, cuticular bars under claws I–III present (Fig. 2).

##### Eggs.

Smooth, deposited in exuvia.

No males were found.

##### Locus typicus.

46°14.74167N, 27°40.27333E; 99 m asl: Romania, Vaslui County, Bârlad town, coppice, lichens (*Xanthoria parietina* (L.) Th. Fr. (1860)) from tree.

##### Etymology.

This new species is named after the Berladnici, an ancient population with a controversial origin (most probably Slavs) who previously lived in the area of the present Bârlad town.

##### Type depositories.

Holotype (female; slide: P8-8) and 29 paratypes (females) and 1 exuvium with eggs (slides: P8-4, P8-5, P8-6, P8-9, P8-13, P8-14, P8-15, P8-17, P8-19) are preserved at the Department of Animal Taxonomy and Ecology, A. Mickiewicz University in Poznań, Umultowska 89, 61–614 Poznań, Poland. Additionally, 14 paratypes (females) and 1 exuvium with eggs (slides: P8-1, P8-3, P8-16, P8-18) are deposited at Natural History Museum of “Alexandru Ioan Cuza” University from Iași (Bd. Independentei No.16, 700101), 4 paratypes (females; slides: P8-7, P8-12) are deposited at collection of Binda and Pilato (Museum of the Department of Animal Biology “Marcello La Greca”, University of Catania, Italy) and 5 paratypes (females; slides: P8-2, P8-10, P8-11) are deposited at the Natural History Museum, University of Copenhagen Universitetsparken 15, DK-2100 Copenhagen, Denmark.

**Figures 1.**
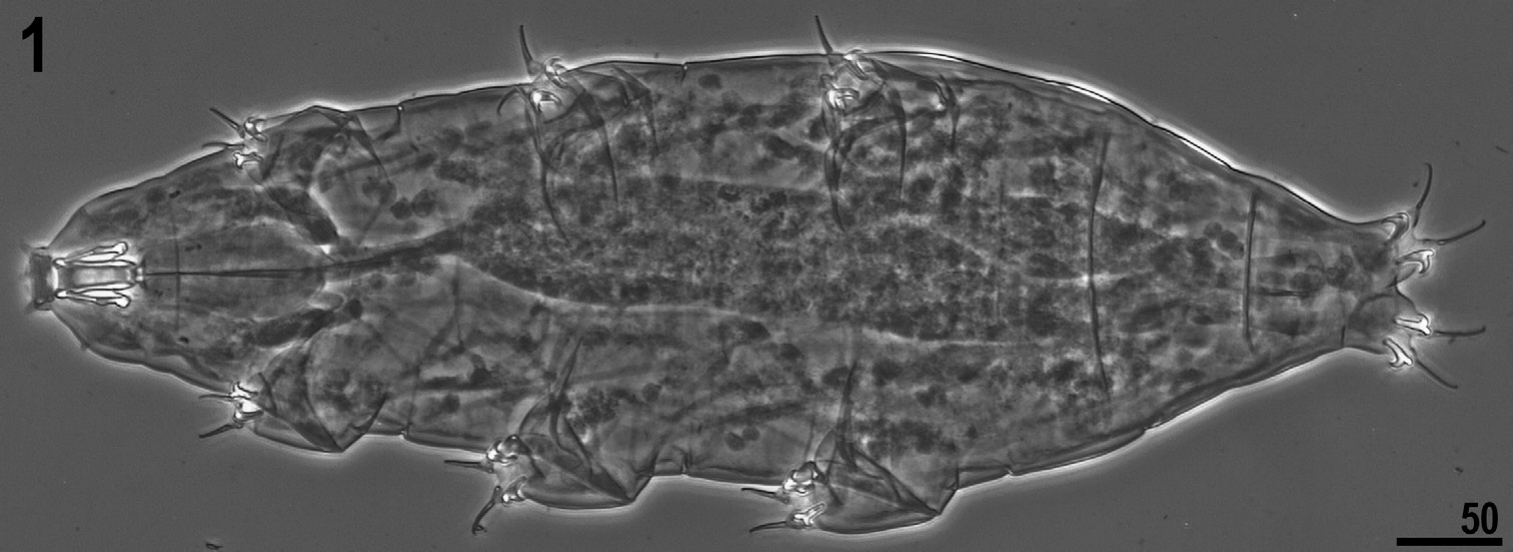
*Milnesium berladnicorum* sp. n. Habitus (ventral view).

**Figures 2–6.**
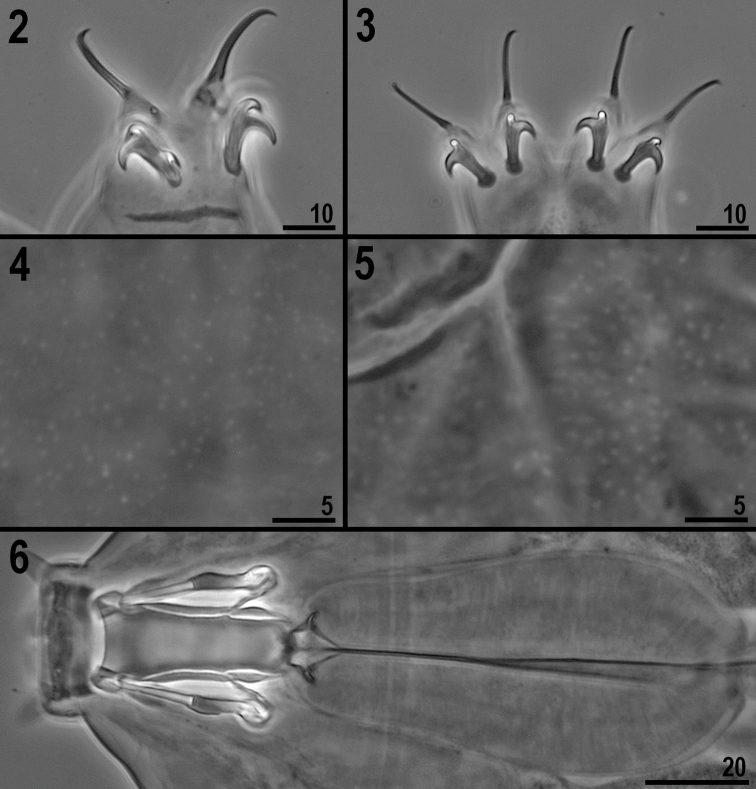
*Milnesium berladnicorum* sp. n.: **2** claws III **3** claws IV **4** sculpture on dorsal cuticle above II–III pair of legs **5** sculpture on dorsal cuticle above IV pair of legs **6** buccal apparatus (ventral view).

**Figure 7.**
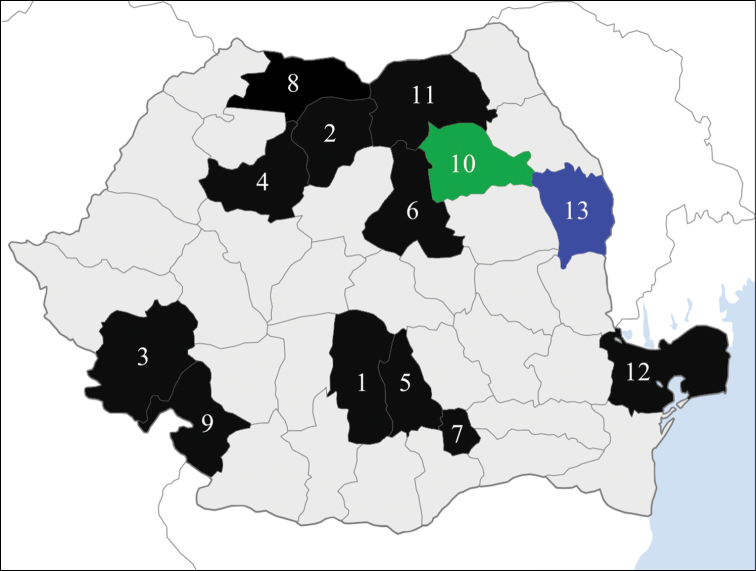
The administrative map of Romania with 13 highlighted counties in which species of the genus *Milnesium* were reported: *Milnesium tardigradum* sensu lato (according with Rudescu 1964; see Discussion): **1** Argeş **2** Bistriţa-Năsăud **3** Caraş-Severin **4** Cluj **5** Dâmboviţa **6** Harghita **7** Ilfov County and Bucharest City **8** Maramureş **9** Mehedinţi **11** Suceava **12** Tulcea. *Milnesium granulatum* and *Milnesium asiaticum* (according to Ciobanu et al. 2014): **10** Neamţ (in green). *Milnesium berladnicorum* sp. n. (present study): **13** Vaslui (in blue). Map outline according to Wikipedia: http://ro.wikipedia.org/wiki/Fi%C8%99ier:Romania_counties_blank_big.png

**Table 1.** Measurements and *pt* values of selected morphological structures of fifteen females from the type population of *Milnesium berladnicorum* sp. n.

CHARACTER	N	RANGE						MEAN		SD		Holotype	
		µm			*pt*			µm	*pt*	µm	*pt*	µm	*pt*
Body length	15	400	–	734	*1557*	–	*1942*	622	*1742*	107	*127*	630	*1619*
Peribuccal papillae length	15	4.0	–	7.9	*14.8*	–	*20.5*	6.2	*17.4*	1.2	*1.6*	7.0	*18.0*
Lateral papillae length	15	3.6	–	6.9	*12.7*	–	*17.9*	5.2	*14.5*	1.0	*1.6*	5.6	*14.4*
Buccal tube													
Length	15	24.7	–	41.5		–		35.7	–	5.3	–	38.9	–
Stylet support insertion point	15	17.2	–	28.3	*66.6*	–	*71.2*	24.6	*69.2*	3.6	*1.3*	27.4	*70.4*
Anterior width	15	8.9	–	17.8	*35.0*	–	*47.1*	14.8	*41.3*	3.0	*3.4*	16.4	*42.2*
Standard width	15	7.8	–	14.7	*30.6*	–	*38.9*	12.0	*33.5*	2.2	*2.3*	12.7	*32.6*
Posterior width	12	7.2	–	13.6	*27.7*	–	*36.0*	11.0	*30.3*	1.8	*2.2*	11.5	*29.6*
Standard width/length ratio	15	31%	–	39%		–		33%		2%	–	33%	
Posterior/anterior width ratio	12	69%	–	79%		–		73%		3%	–	70%	
Claw 1 lengths													
External primary branch	14	10.0	–	18.0	*40.5*	–	*47.6*	15.6	*43.9*	2.5	*2.2*	17.6	*45.2*
External base + secondary branch	14	10.2	–	15.9	*32.9*	–	*45.3*	13.9	*39.3*	2.0	*3.0*	14.6	*37.5*
External spur	0		–			–		–	–	–	–	–	–
Internal primary branch	14	9.9	–	18.1	*38.1*	–	*45.6*	14.8	*41.8*	2.3	*2.2*	15.6	*40.1*
Internal base + secondary branch	14	9.9	–	15.0	*33.9*	–	*41.7*	13.1	*37.3*	1.8	*2.2*	13.2	*33.9*
Internal spur	9	3.0	–	4.7	*9.0*	–	*12.5*	4.0	*11.2*	0.6	*1.1*	3.5	*9.0*
Claw 2 lengths													
External primary branch	15	11.5	–	20.0	*43.3*	–	*53.5*	17.2	*48.4*	2.6	*3.2*	20.0	*51.4*
External base + secondary branch	15	10.4	–	16.2	*36.9*	–	*44.4*	14.4	*40.6*	1.9	*2.2*	15.8	*40.6*
External spur	0		–			–		–	–	–	–	–	–
Internal primary branch	15	11.4	–	18.7	*39.9*	–	*51.8*	16.1	*45.4*	2.3	*3.1*	17.2	*44.2*
Internal base + secondary branch	15	9.8	–	15.8	*34.4*	–	*42.5*	13.5	*38.0*	2.0	*2.7*	14.4	*37.0*
Internal spur	6	2.6	–	5.9	*10.2*	–	*15.6*	4.1	*12.4*	1.2	*2.2*	?	?
Claw 3 lengths													
External primary branch	15	11.1	–	20.5	*44.9*	–	*54.8*	17.6	*49.5*	2.8	*3.1*	17.9	*46.0*
External base + secondary branch	15	9.9	–	16.9	*37.8*	–	*44.5*	14.6	*40.9*	2.2	*2.1*	16.4	*42.2*
External spur	0		–			–		–	–	–	–	–	–
Internal primary branch	15	10.6	–	20.0	*39.4*	–	*53.7*	16.5	*46.4*	2.8	*4.5*	15.5	*39.8*
Internal base + secondary branch	13	9.9	–	17.6	*33.6*	–	*45.6*	13.7	*38.9*	2.2	*3.2*	15.2	*39.1*
Internal spur	5	3.2	–	5.3	*10.6*	–	*14.0*	4.3	*12.0*	0.8	*1.4*	?	?
Claw 4 lengths													
Anterior primary branch	15	15.0	–	27.0	*57.9*	–	*74.8*	22.7	*63.8*	3.4	*4.7*	24.8	*63.8*
Anterior base + secondary branch	15	11.5	–	20.2	*40.0*	–	*50.0*	16.7	*46.9*	2.6	*3.0*	18.5	*47.6*
Anterior spur	0		–			–		–	–	–	–	–	–
Posterior primary branch	15	14.2	–	25.8	*54.2*	–	*70.4*	22.1	*62.0*	3.4	*4.6*	24.0	*61.7*
Posterior base + secondary branch	15	11.3	–	19.6	*38.2*	–	*52.2*	16.5	*46.2*	2.9	*4.0*	17.4	*44.7*
Posterior spur	0		–			–		–	–	–	–	–	–

### Differential diagnosis

Due to the sculptured cuticle, *Milnesium berladnicorum* sp. n. belongs to the *granulatum* group (Michalczyk et al. 2012a, 2012b). The new species differs from all other species in the *granulatum* group by the presence of a unique claw configuration [2-3]-[2-2] that is not present in any other species in this group. Besides the claw configuration, the new species differs from:

1. *Milnesium alabamae* Wallendorf and Miller, 2009: by having a different cuticular sculpture (sparse pseudopores on the cuticle which do not form a true reticulum in *Milnesium berladnicorum* sp. n. *vs* a finely punctuated (probably pseudopores) cuticle arranged in bands on caudal segments in *Milnesium alabamae*), a different claw configuration ([2-3]-[2-2] in *Milnesium berladnicorum* sp. n. *vs* [3-3]-[3-3] in *Milnesium alabamae*), the presence of accessory points on primary branches and by presence of eyes.

2. *Milnesium beasleyi* Kaczmarek, Jakubowska and Michalczyk, 2012: by having a different claw configuration ([2-3]-[2-2] in *Milnesium berladnicorum* sp. n. *vs.* [2-3]-[3-2] in *Milnesium beasleyi*), a different posterior/anterior width ratio (69%–79% in *Milnesium berladnicorum* sp. n. *vs* 90%–96% in *Milnesium beasleyi*) and stylet supports inserted in a more posterior position (*pt=66.6*–*71.2* in *Milnesium berladnicorum* sp. n. *vs pt=61.6*–*65.6* in *Milnesium beasleyi*).

3. *Milnesium granulatum* (Ramazzotti, 1962): by having a different cuticular sculpture (sparse pseudopores on the cuticle which do not form a true reticulum in *Milnesium berladnicorum* sp. n. *vs* a reticular sculpture in *Milnesium granulatum*) and different claw configuration ([2-3]-[2-2] in *Milnesium berladnicorum* sp. n. *vs* [3-3]-[3-3] in *Milnesium granulatum*).

4. *Milnesium katarzynae* Kaczmarek, Michalczyk and Beasley, 2004: by having a different cuticular sculpture (sparse pseudopores on the cuticle which do not form a true reticulum in *Milnesium berladnicorum* sp. n. *vs* a reticular sculpture in *Milnesium katarzynae*), a different claw configuration ([2-3]-[2-2] in *Milnesium berladnicorum* sp. n. *vs* [2-2]-[2-2] in *Milnesium katarzynae*), larger body size (400–734 µm in *Milnesium berladnicorum* sp. n. *vs* 285.0–294.5 µm in *Milnesium katarzynae*), stylet supports inserted in a more anterior position (*pt=66.6*–*71.2* in *Milnesium berladnicorum* sp. n. *vs pt=73.3*–*78.3* in *Milnesium katarzynae*) and by the presence of eyes.

5. *Milnesium krzysztofi* Kaczmarek and Michalczyk, 2007: by having a different cuticular sculpture (sparse pseudopores on the cuticle which do not form a true reticulum in *Milnesium berladnicorum* sp. n. *vs* dorsal cuticle with pseudopores arranged in a fine reticular design in *Milnesium krzysztofi*), a different claw configuration ([2-3]-[2-2] in *Milnesium berladnicorum* sp. n. *vs* [2-3]-[3-2] in *Milnesium krzysztofi*) and by presence of eyes.

6. *Milnesium lagniappe* Meyer, Hinton and Dupré, 2013: by the presence of six peribuccal lamellae (four in *Milnesium lagniappe*), a different cuticular sculpture (sparse pseudopores on the cuticle which do not form a true reticulum in *Milnesium berladnicorum* sp. n. *vs* nine dorsal and lateral sculptured bands bearing a reticulated pattern of polygons in *Milnesium lagniappe*), a different claw configuration ([2-3]-[2-2] in *Milnesium berladnicorum* sp. n. *vs.* [2-3]-[3-2] in *Milnesium lagniappe*), a smaller anterior width of buccal tube (8.9–17.8 µm in *Milnesium berladnicorum* sp. n. *vs* 20.7–25.1 µm in *Milnesium lagniappe*), a smaller standard width of the buccal tube (7.8–14.7 μm in *Milnesium berladnicorum* sp. n. *vs.* 19.4–23.6 μm in *Milnesium lagniappe*), a smaller posterior width of the buccal tube (7.2–13.6 µm in *Milnesium berladnicorum* sp. n. *vs* 18.9–23.2 µm in *Milnesium lagniappe*), a smaller posterior/anterior width ratio (69%–79% in *Milnesium berladnicorum* sp. n. *vs* 86%–99% in *Milnesium lagniappe*) and a smaller standard width/length ratio (31%–39% in *Milnesium berladnicorum* sp. n. *vs* 63%–78% in *Milnesium lagniappe*).

7. *Milnesium reticulatum* Pilato, Binda and Lisi, 2002: by the lack of dorsal gibbosities, the presence of six peribuccal lamellae (four in *Milnesium reticulatum*), a different claw configuration ([2-3]-[2-2] in *Milnesium berladnicorum* sp. n. *vs* [2-3]-[3-2] in *Milnesium reticulatum*) and slightly larger body length (400–734 μm in *Milnesium berladnicorum* sp. n. *vs.* 270–405 μm in *Milnesium reticulatum*).

Because of the claw configuration [2-3]-[2-2], *Milnesium berladnicorum* sp. n. is similar to *Milnesium almatyense* Tumanov, 2006 (Michalczyk et al. 2012a, 2012b) but differs by having a sculptured dorsal cuticle and by presence of eyes.

## Discussion

Until 1990, the genus *Milnesium* Doyère, 1840 was considered as monotypic with only one described cosmopolitan species, *Milnesium tardigradum* Doyère, 1840. In 1990, Binda and Pilato described a second species, *Milnesium brachyungue* from Chile. Later, additional species in the genus *Milnesium* were described sporadically up to 2006 when Tumanov published the first, but partial, revision of the genus *Milnesium* and described five new species (Tumanov 2006). In 2012, the genus *Milnesium* was redescribed in more detail by Michalczyk et al. (2012a, 2012b), and the nominal species *Milnesium tardigradum tardigradum* sensu stricto Doyère,1840 obtained a clear and definitive diagnosis.

At present the genus *Milnesium* consists of 21 species and one subspecies (Degma et al. 2014), which have been divided into two groups (based on the presence/absence of a sculptured cuticle): *tardigradum* and *granulatum* (Michalczyk et al. 2012a, 2012b). Due to the sculptured cuticle, *Milnesium berladnicorum* sp. n. belongs to the *granulatum* group. Including the new species, the *granulatum* group now consists of eight species: *Milnesium alabamae* Wallendorf and Miller, 2009 (from USA), *Milnesium berladnicorum* sp. n. (from Romania), *Milnesium granulatum* (Ramazzotti, 1962) (from Chile, Italy, Romania and USA), *Milnesium katarzynae* Kaczmarek et al., 2004 (from Costa Rica and China), *Milnesium krzysztofi* Kaczmarek and Michalczyk, 2007 (Costa Rica and Peru), *Milnesium reticulatum* Pilato et al., 2002 (Seychelles), *Milnesium beasleyi* Kaczmarek et al., 2012 (Turkey) and *Milnesium lagniappe* Meyer et al., 2013 (USA) (Ramazzotti 1962; Pilato et al. 2002; Kaczmarek et al. 2004; Kaczmarek and Michalczyk 2007; Wallendorf and Miller 2009; Kaczmarek et al. 2012; Michalczyk et al. 2012a, 2012b; Meyer et al. 2013; Kaczmarek et al. 2014; Bartels et al. 2014). Thus, *granulatum* group is equivalent to *ca.* 39% of all known *Milnesium* taxa and the “sculptured species” are distributed around the World.

According to Rudescu (1964), *Milnesium tardigradum* sensu lato Doyère, 1840 was firstly reported in Romanian territory by Botezat (1903) in the area of Suceava County (Austro-Hungarian Empire at the time). Later, it was reported numerous times at different Romanian localities by: Rodewald (1936), Iharos (1937, 1940, 1962), Péterfi (1956), Botoşăneanu and Negrea (1961), and Rudescu (1964). However, based on modern literature, all these records should be considered as dubious and need verification (Michalczyk et al. 2012a, 2012b). This is now even more necessary due to the discovery in 2014 of two other *Milnesium* species in Romania (Ciobanu et al. 2014): **a)** *Milnesium asiaticum* Tumanov, 2006, previously known only from three localities in Kyrgyzstan (Tumanov 2006), Spitsbergen (Kaczmarek et al. 2012b) and Estonia (Zawierucha et al. 2014); and **b)** *Milnesium granulatum* (Ramazzotti, 1962) previously known only from three localities in Chile, Italy and USA (McInnes 1994; Bartels et al. 2014) (see map above).

Including the new species described here, the total number of valid tardigrade taxa recorded in Romania is 128, with three valid *Milnesium* species (not including *Milnesium tardigradum tardigradum* sensu stricto, which requires confirmation of presence in Romania).

## Acknowledgments

The authors want to thank Prof. Diane Nelson of East Tennessee State University for help in improving of the English in the manuscript. We are also grateful to anonymous

reviewers for valuable remarks. This work was partially funded by the Prometeo Project of the Secretariat for Higher Education, Science, Technology and Innovation of the Republic of Ecuador. Studies have been partially conducted in the framework of activities of BARg (Biodiversity and Astrobiology Research group) at the Adam Mickiewicz University in Poznań, Poland.

## Supplementary Material

XML Treatment for Milnesium berladnicorum
